# Characteristics of women who underwent one or more previous cesarean sections according to Nascer no Brasil

**DOI:** 10.11606/s1518-8787.2023057004819

**Published:** 2023-11-08

**Authors:** Marina Barreto Alvarenga, Silvana Granado Nogueira da Gama, Marcos Nakamura-Pereira

**Affiliations:** I Fundação Oswaldo Cruz Escola Nacional de Saúde Pública Sérgio Arouca Programa de Epidemiologia em Saúde Pública Rio de Janeiro RJ Brasil Fundação Oswaldo Cruz. Escola Nacional de Saúde Pública Sérgio Arouca. Programa de Epidemiologia em Saúde Pública, Rio de Janeiro, RJ, Brasil; II Fundação Oswaldo Cruz Escola Nacional de Saúde Pública Sérgio Arouca Departamento de Epidemiologia e Métodos Quantitativos em Saúde Rio de Janeiro RJ Brasil Fundação Oswaldo Cruz. Escola Nacional de Saúde Pública Sérgio Arouca. Departamento de Epidemiologia e Métodos Quantitativos em Saúde, Rio de Janeiro, RJ, Brasil; III Fundação Oswaldo Cruz Escola Nacional de Saúde Pública Sérgio Arouca Programa de Epidemiologia em Saúde Pública Rio de Janeiro RJ Brasil Fundação Oswaldo Cruz. Escola Nacional de Saúde Pública Sérgio Arouca. Programa de Epidemiologia em Saúde Pública, Rio de Janeiro, RJ, Brasil; IV Instituto Fernandes Figueira Rio de Janeiro RJ Brasil Instituto Fernandes Figueira, Rio de Janeiro, RJ, Brasil

**Keywords:** Delivery, Obstetric, Vaginal Birth After Cesarean, Natural Childbirth, Cesarean Section, Maternal Health

## Abstract

**OBJECTIVE:**

To descriptively analyze Brazilian parturient women who underwent previous cesarean section and point out the factors associated with Vaginal Birth After Cesarean (VBAC) in Brazil.

**METHODS:**

The study used data from women with one, two, or three or more cesarean sections from the survey *Nascer no Brasil* (Birth in Brazil). Differences between categories were assessed through the chi-square test (χ^2^). Variables with significant differences (p < 0.05) were incorporated into logistic regression.

**FINDINGS:**

Out of the total of 23,894 women, 20.9% had undergone a previous cesarean section. The majority (85.1%) underwent another cesarean section, with 75.5% occurring before the onset of labor. The rate of Vaginal Birth After Cesarean (VBAC) was 14.9%, with a success rate of 60.8%. Women who underwent three or more cesarean sections displayed greater social vulnerability. The chances of VBAC were higher among those who opted for a vaginal birth towards the end of gestation, had a prior vaginal birth, underwent labor induction, were admitted with over 4 centimeters of dilation, and without partner. Receiving care from the private health care system, having two or more prior cesarean sections, obstetric complications, and deciding on cesarean delivery late in gestation reduced the chances of VBAC. Age group, educational background, prenatal care adequacy, and the reason for the previous cesarean section did not result in significant differences.

**CONCLUSION:**

The majority of women who underwent a previous cesarean section in Brazil are directed towards another surgery, and a higher number of cesarean sections is linked to greater social inequality. Factors associated with VBAC included choosing vaginal birth towards the end of gestation, having had a previous vaginal birth, higher cervical dilation upon admission, induction, assistance from the public health care system, absence of obstetric complications, and without a partner. Efforts to promote VBAC are necessary to reduce overall cesarean rates and their repercussions on maternal and child health.

## INTRODUCTION

In Brazil, since 2009^1^, most births have occurred via cesarean section. Despite all the public policies implemented in the country in recent years^2^, data from the Ministry of Health indicate that the national rate was 57.2%^6^ in 2020. According to the World Health Organization (WHO), cesarean rates higher than 10% to 15% are not associated with a reduction in maternal and neonatal mortality, and there is no evidence of the benefits of surgery for women without clinical recommendations. Furthermore, surgical procedures entail more immediate and long-term risks for the health of women and their children, including possible future pregnancies^7^.

For women experiencing their first pregnancy in Brazil, the likelihood of a vaginal birth is already relatively low, and for those who have undergone prior cesarean sections, these chances are even more unlikely^8^. The notion of “once a cesarean, always a cesarean,” originating as far back as 1916^11^, is still dominant within the obstetric culture of the country, despite the plethora of scientific evidence highlighting the safety of Vaginal Birth After Cesarean (VBAC). The choice regarding the type of delivery in subsequent pregnancies following a cesarean section becomes a significant concern, given the potential risks linked to repetitive surgeries within the same region, encompassing an increased chance of adhesions, hemorrhages, and atypical placental positioning (placenta previa and accreta)^12^, which can result in severe maternal complications, including death.

In Brazil, accessible data concerning maternal and perinatal health after cesarean sections is scarce, highlighting the need for additional research to achieve a comprehensive grasp of the subject. The objective of this study is to conduct a descriptive analysis of Brazilian parturients who have undergone a previous cesarean section and are experiencing a subsequent pregnancy, as well as to identify factors associated with Vaginal Birth After Cesarean (VBAC) in Brazil.

## METHODS

This study uses data from *Nascer no Brasil* (Birth in Brazil), a national survey on childbirth and delivery, comprising postpartum women and their newborns. The survey was conducted from February 2011 to October 2012, with a representative sample from all five macroregions of the country, including residents from both capital and non-capital areas, across private, public, and mixed health care services. Data collection involved interviews during hospitalization, data extraction from medical records, prenatal care cards (when available), and two phone calls after hospital discharge. The data were collected through a complex sampling approach encompassing 266 hospitals^15,16^.

This study centers on a cohort of 4,987 women who had experienced a previous cesarean section. This group excludes nulliparous and multiparous women who have solely undergone vaginal deliveries (Figure 1). Sociodemographic and obstetric characteristics were evaluated, stratified by the number of cesarean surgeries (one, two, three, or more) and the type of delivery in the current pregnancy, whether vaginal (VBAC) or repeat cesarean section.

**Figure:**
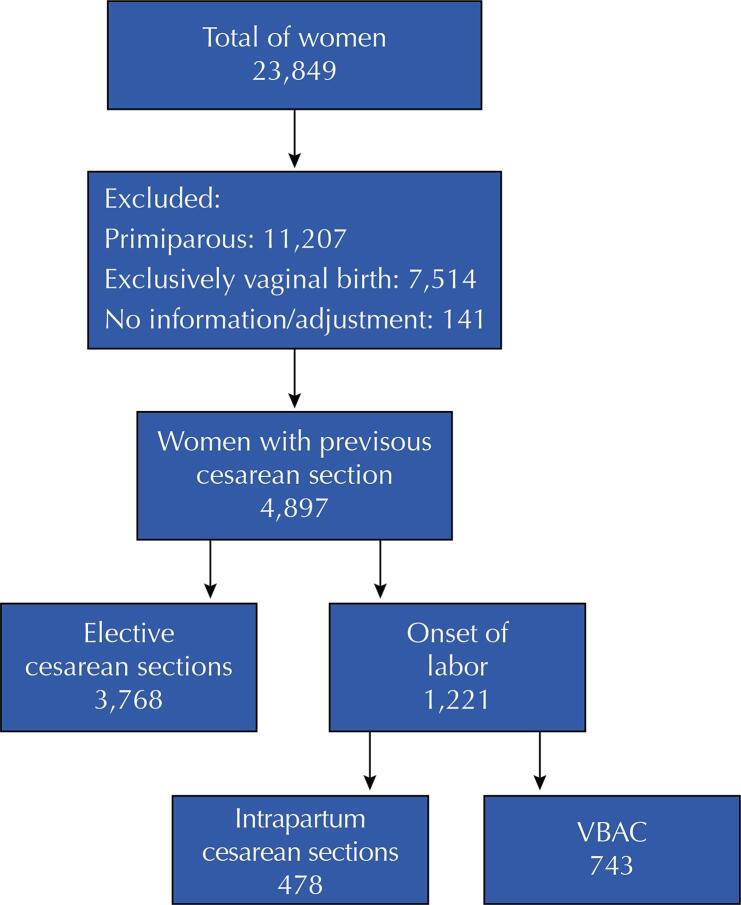
Flowchart of women who underwent one or more cesarean sections according to Birth in Brazil. Period: 2011–2012. Source: Birth in Brazil survey. VBAC: Vaginal Birth After Cesarean.

Source: Inquérito Nascer no Brasil (Birth in Brazil survey).

Selected sociodemographic variables included: region of occurrence (North, Northeast, Central-West, Southeast, and South); city location (capital, non-capital); funding for delivery (Unified Health System [SUS], private); age group (12-19, 20-34 years, > 35); race/ethnicity according to the Brazilian Institute of Geography and Statistics (Instituto Brasileiro de Geografia e Estatística [IBGE]) (White, Black, Mixed, Yellow, Indigenous); educational background in years (≤ 7, 8-10, ≥ 11); marital status (with or without a partner); and grouped social class (A/B, C, or D/E) according to the Brazilian Association of Research Companies (Associação Brasileira de Empresas de Pesquisa [ABIPEME])^17^.

Selected obstetric factors included: type of delivery in the current pregnancy (vaginal, cesarean); cesarean section based on labor (cesarean before labor, intrapartum cesarean); previous vaginal birth; consistent prenatal and delivery care provider (yes, no); gestational age (premature – up to 36 weeks, term – 37 to 41 weeks, post-term – ≥ 42 weeks); labor induction (induced, spontaneous); preferred mode of delivery at the start of pregnancy and the end of pregnancy (vaginal, cesarean, no preference).

Prenatal care adequacy (no prenatal care, inadequate, adequate) was based on the study by Domingues et al^18^. Prenatal care was considered adequate when initiated within the first 12 weeks of gestation, with a minimum of six consultations, documentation of at least one result for each routine prenatal test in the pregnancy record, and guidance provided to the reference maternity facility.

Regarding cervical dilation at the time of hospitalization (£ 3 centimeters, 4-5 centimeters, ≥ 6 centimeters), it was noted that 95% of women lacked this information in their medical records underwent a cesarean section before the onset of labor. These cases were thus categorized under “cervical dilation under 3 centimeters.” The remaining 5% were evenly distributed among the other categories. In terms of the labor success rate, the calculation was based on the percentage of vaginal deliveries among women who experienced labor.

Emergencies or obstetric complications encompassed conditions like hypertensive syndromes, diabetes, or any clinical and obstetric situation that could potentially lead to obstetric emergencies before delivery, including instances such as premature placental detachment, placenta previa, HIV infection, and other relevant factors^10,19^. Data were obtained from medical records, except for those related to race/ethnicity, education, marital status, social class, and reasons for the most recent cesarean section. Prenatal care adequacy was evaluated based on prenatal care cards. The current indication for cesarean sections comprised up to three potential options for each patient, as determined by the attending obstetrician.

Descriptive statistical analysis was carried out to present both absolute and relative frequencies of sociodemographic and obstetric variables, categorized by the number of prior cesarean sections and the type of delivery of choice for the ongoing pregnancy. Disparities between categories were evaluated employing the chi-square test (χ^2^), with a significance level set at p < 0.05. Variables exhibiting statistically significant differences during the descriptive analyses were progressively integrated into a hierarchical generalized linear model, starting with sociodemographic attributes and subsequently incorporating further obstetric characteristics. The statistical adjustment could nullify the effect power of such variables. The R software, version 1.2.1335, was used for the analyses.

### Ethical Aspects

The study was approved by the Ethics Committee of the Sergio Arouca National School of Public Health, Oswaldo Cruz Foundation (Escola Nacional de Saúde Pública Sergio Arouca, Fundação Oswaldo Cruz [ENSP/Fiocruz]), under protocol CAAE 50697621.4.0000.5240, with approval number: 4.950.262.

## RESULTS

Out of the overall cohort of 23,894 women enrolled in the *Nascer no Brasil* survey, a total of 4,987 individuals (20.9%) had undergone at least one prior cesarean section (Figure). Table 1 shows that the predominant proportion of women who received health care funded by the Unified Health System (SUS) (71.8%) were between 20-34 years old (76.8%), identified themselves as mixed (52.4%), had attained more than 11 years of education (51.6%), cohabited with a partner (86.6%), and were categorized within social class C (49.3%).

**Table 1:** Frequency table. Sociodemographic characteristics of women in Brazil who have undergone prior cesarean sections. Breakdown according to the count of previous cesarean sections, type of delivery in the ongoing pregnancy, and the percentage of Vaginal Birth After Cesarean (VBAC) within each category. Period: 2011-2012. Source: Birth in Brazil survey.

Variables	All women who underwent previous cesarean sections		1 previous cesarean section		2 previous cesarean sections		≥3 previous cesarean sections		p-value	VBAC		% VBAC	Repeat cesarean sections		p-value
						
Sociodemographic factors	n	%	n	%	n	%	n	%		n	%		n	%	
Total	4,987	100	3817	100	948	100	222	100		743	100	14.9	4,246	100	
Region															
North	426	8.6	313	8.2	104	10.9	10	4.5		83	11.2	19.5	343	8.1	
Northeast	1,194	24.0	952	25.0	219	23.1	22	9.8		148	19.9	12.4	1,045	24.6	
Midwest	368	7.4	256	6.7	84	8.9	27	12.4	< 0.001	47	6.4	12.8	320	7.6	0.15
Southeast	2,281	45.7	1,747	45.8	418	44.1	117	52.5		343	45.3	15.0	1,938	45.6	
South	718	14.4	549	14.4	123	13.0	46	20.7		120	16.2	16.7	598	14.1	
City															
Capital	1,831	36.7	1,391	36.4	354	37.4	86	38.7	0.78	309	41.6	16.9	1,523	64.1	0.13
Countryside	3,156	63.3	2,426	63.6	592	62.6	136	61.3		434	58.4	13.8	2,723	35.9	
Hospital															
SUS	3,584	71.8	2,629	68.9	758	79.9	196	88.2	< 0.001	713	96.1	24.6	2,871	67.6	
Private	1,404	28.2	1,118	31.1	190	21.1	26	11.8		29	3.9	2.0	1,375	32.4	
Age group															
10–19	252	5.1	224	5.9	22	2.3	6	2.6		60	8.1	23.8	192	4.5	
20–34	3,834	76.8	2,933	76.8	740	78.0	161	71.7	< 0.001	602	81	15.7	3,233	76.1	< 0.001
35 or older	901	18.1	660	17.3	186	29.7	55	25.7		81	10.9	9.0	821	19.4	
Race/ethnicity															
White	1,935	38.8	1,52	39.8	332	35.0	82	37.1		253	34.1	13.1	1,682	39.6	
Black	375	7.5	286	7.5	62	6.6	27	12.1		70	9.4	18.7	305	7.2	
Mixed	2,615	52.4	1,966	51.5	536	56.5	112	50.6	0.02	407	54.8	15.6	2,208	52	0.12
Asian	53	1.1	36	0.9	16	1.7	1	0.3		10	1.3	18.9	43	1.0	
Indigenous	11	0.2	9	0.2	2	0.3	0	0		3	0.5	27.3	8	0.2	
Education															
Up to 7 years	1,281	25.7	847	22.2	321	33.9	113	50.8		290	38.9	22.6	993	23.4	
8–10 years	1,134	22.7	827	21.7	251	26.5	56	25.4	< 0.001	207	27.9	18.3	927	21.8	< 0.001
11 or older	2,572	51.6	2,143	56.1	376	39.6	53	23.8		246	33.2	9.6	2,326	54.8	
Marital status															
Single	669	13.4	477	12.5	151	16.0	40	18.0	0.04	150	20.3	22.4	518	12.2	< 0.001
Married/in a relationship	4,317	86.6	3,337	87.5	797	84.0	182	82.0		590	79.7	13.7	3,272	87.8	
Financial status category according to ABIPEME															
A/B	1,621	32.5	1,306	33.2	259	27.3	56	25.1		117	15.7	7.2	1,505	35.5	
C	2,457	49.3	1,861	48.8	481	50.8	115	51.6	< 0.001	424	57	17.3	2,035	47.9	< 0.001
D/E	909	18.2	649	17.0	208	21.9	51	23.3		202	27.3	22.2	706	16.6	

SUS: *Sistema Único de Saúde* (Unified Health System); ABIPEME: *Associação Brasileira de Empresas de Pesquisa*.

Source: *Inquérito Nascer no Brasil* (Birth in Brazil survey).

Certain categories demonstrated an escalation in proportions as the number of surgeries increased. For instance, Black women constituted 7.5% of those who had undergone one previous cesarean section, which reached 12% among those with three or more cesarean sections. Similarly, individuals falling within the lower educational status (with up to 7 years of education) rose from 22.2% to 50.8%. Funding through the Unified Health System (SUS) also increased from 68.9% to 88.2%. Furthermore, the percentages of women over 35 years old, without a partner, and belonging to social classes C, D, and E also had an increase.

Taking all women into account, the rate of Vaginal Birth After Cesarean (VBAC) stood at 14.9%. Notably, the SUS accounted for 96.1% of VBAC cases, and, among deliveries performed within the public system involving a history of previous cesarean sections, 24.6% resulted in vaginal births. In terms of region, city type (capital or non-capital), and race/ethnicity, no statistically significant differences were observed regarding the type of delivery. However, when scrutinized by category, higher proportions of VBAC were apparent among women from the North region (19.5%), residents of capital cities (16.9%), indigenous women (27.3%), those aged 12-19 years old (23.8%), individuals with under 7 years of education (22.6%), those in social classes C and D (22.2%), and those without a partner (22.4%).

Table 2 provides insight into the obstetric characteristics of women who underwent previous cesarean sections. The majority of these women had undergone a single cesarean section (76.5%). Among this group, 81.3% subsequently experienced another cesarean section, with 75.5% occurring before the onset of labor. The percentage of cesarean delivery in the ongoing pregnancy reached 97.0% among women who had undergone two or more previous cesarean surgeries.

**Table 2:** Frequency table. Obstetric characteristics of women in Brazil who have undergone prior cesarean sections. Breakdown according to the count of previous cesarean sections, type of delivery in the ongoing pregnancy, and the percentage of Vaginal Birth After Cesarean (VBAC) within each category. Period: 2011-2012. Source: Birth in Brazil survey.

Variables	All women who underwent previous cesarean sections		1 previous cesarean section		2 previous cesarean sections		≥3 previous cesarean sections		p-value	VBAC		% VBAC	Repeat cesarean sections		p-value
						
Sociodemographic factors	n	%	n	%	n	%	n	%		n	%		n	%	
Total	4,987	100	3817	100	948	100	222	100		743	100	14.9	4,246	100	
Number of cesarean sections															
1	3817	76.5	-	-	-	-	-	-	-	714	96.0	18.7	3,104	73.1	
2	948	19.0	-	-	-	-	-	-	-	23	3.1	2.4	926	21.8	< 0.001
3 or more	222	4.5	-	-	-	-	-	-	-	6	0.9	2.7	216	5.1	
Cesarean according to labor															
Cesarean section without labor	3,768	75.5	2,723	71.3	844	89	200	90.0	< 0.001	-	-	-	3,768	88.7	
Intrapartum cesarean section	478	9.6	380	10.0	81	8.9	16	7.2		-	-	-	478	11.3	-
Previous vaginal birth															
Yes	1049	21	840	22	159	16.8	22	22.3	0.04	412	55.6	55.5	636	15.1	< 0.001
No	3932	79	2971	73	789	83.2	78	77.7		328	44.4		3604	84.9	
Labor success rate*															
Vaginal births	743	60.8	714	65.2	23	22.1	6	28.1	-						
Prenatal															
No prenatal	63	1.3	39	1.0	12	1.3	10	4.9		18	2.4	27.2	45	1.1	
Inadequate	2,146	43.0	1,595	41.8	441	46.5	110	49.7	< 0.001	404	54.4	18.2	1,743	41.0	< 0.001
Adequate	2,779	55.7	2,183	57.2	495	52.2	101	45.4		321	43.2	11.2	2,458	57.9	
Same professional in prenatal care and childbirth															
Yes	1,716	34.4	1,378	36.1	293	30.9	44	20.1	< 0.001	31	4.2	1.8	1,685	39.7	
No	3,271	65.6	2,439	63.9	655	69.1	178	79.9		711	95.8	21.7	2,56	60.3	< 0.001
Gestational age															
Premature (≤ 36 wk)	491	9.9	354	9.3	110	11.6	27	12.2		77	10.4	15.7	413	9.7	
Full term (37-41 wk)	4,397	88.1	3,377	88.4	827	87.3	190	85.4	0.15	647	87.2	14.7	3,75	88.3	0.84
≥ 42 weeks	102	2	86	2.3	11	1.1	5	2.4		18	2.7	17.6	84	2	
Occurrence of obstetric complications															
Arterial hypertension	665	13.3	499	13.1	127	13.4	39	17.8	0.24	53	7.2	8.0	612	14.4	< 0.001
Diabetes	538	10.8	404	10.6	100	10.5	34	15.3	0.29	65	8.8	12.1	473	11.1	0.17
Emergencies or complications**	1,183	23.7	889	23.3	222	23.4	72	32.3	0.05	113	15.3	9.6	1,07	25.2	< 0.001
Start of labor***															
Induced	212	4.2	208	5.4	1	0.1	3	1.4	< 0.001	106	14.3	50.0	106	2.4	< 0.001
Spontaneous	1,109	22.2	986	25.8	104	11.1	19	8.6		636	85.6	57.3	473	11.2	
Dilation on admission															
≤ 3 cm	4,271	85.6	3,18	83.3	880	92.8	211	95.0		316	42.8	7	3,955	93.1	
4–5 cm	416	8.4	367	9.6	40	4.2	9	4.1	< 0.001	223	29.9	54	193	4.6	< 0.001
≥ 6 cm	300	6.0	270	7.1	28	3.0	2	0.9		203	27.3	68	97	2.3	
Preference for type of delivery															
Vaginal birth	1,756	35.2	1,426	37.4	254	26.9	74	33.7		404	54.4	23.0	1,352	31.8	
C-section	2,869	57.5	2,114	55.4	636	67.0	118	54.4	< 0.001	292	39.4	10.2	2,576	60.7	< 0.001
No preference	364	7.3	277	7.2	58	6.1	29	12.9		46	6.2	12.6	317	7.5	
Decision at the end of pregnancy															
Vaginal birth	605	12.1	572	15.0	30	3.2	3	1.3		373	50.5	61.6	232	12.1	
C-section	3,239	64.9	2,247	58.9	798	84.1	194	87.3	< 0.001	90	12.1	2.8	3,15	64.9	< 0.001
No preference	1,143	22.9	998	26.2	120	12.7	25	11.4		280	37.7	25.1	864	22.9	

Source: *Inquérito Nascer no Brasil* (Birth in Brazil survey).

The percentage of vaginal births among those who went into labor was 60.8%, and this was considered the VBAC success rate within the sample (Figure). Women admitted during active labor had a higher rate of VBAC, whether with cervical dilation of 4-5 centimeters (54%) or exceeding 6 centimeters (68%). Additionally, 55.5% of women who achieved VBAC had a history of a previous vaginal birth in addition to cesarean section.

A significant share of women expressed a preference for another cesarean section at the outset of pregnancy (57.5%), a proportion that increased further among women with two previous cesarean sections (67%). Across all groups, there was an upward trend in the inclination towards cesarean sections as pregnancy progressed, culminating in an overall rate of 64.9%. The category with the highest VBAC percentage was that of women who maintained a preference for vaginal birth throughout the course of prenatal care (61.6%).

The subset of women who underwent three or more previous cesarean sections showed a higher frequency of pregnancies either devoid of prenatal care (4.9%) or characterized by inadequate prenatal care (49.7%). They also experienced a higher incidence of emergencies or complications (32.3%). Furthermore, this group had a lower percentage of follow-up with the same health care professional for both prenatal care and delivery (20.1%), an elevated rate of repeat cesarean sections without labor (90%), consequently leading to admissions with less than 3 cm of cervical dilation (95%).

When inquired about the reasons for their previous cesarean section, “lack of dilation, baby too large, or baby not engaging/descending” emerged as the most prevalent justification (42.7%). This was followed by reasons such as high blood pressure (10.5%), breech presentation (8%), fear of labor pain (4.7%), and prolonged pregnancy (4.3%). The reasons for the current cesarean section, as indicated by data from the obstetrician in the medical records, are detailed in Table 3. The previous cesarean section was cited as the rationale for the current surgery in 44.1% of cases, reaching a notable 73.6% among women with three prior cesarean sections. Other noteworthy prevalent reasons included cephalopelvic disproportion (9.5%) and hypertensive syndromes (7.6%).

**Table 3:** Reasons indicated by the obstetrician for cesarean sections in current pregnancies among women with previous cesarean sections in Brazil. Period: 2011-2012. Source: Birth in Brazil survey.

Reasons for cesarean sections	All women who underwent previous cesarean sections		1 previous cesarean section		2 previous cesarean sections		≥ 3 previous cesarean sections	
			
	n	%	n	%	n	%	n	%
Previous cesarean sections	2,256	44.1	1,398	45.2	683	70.1	173	73.6
Cephalopelvic disproportion	490	9.5	452	14.6	25	2.6	6	2.6
Hypertensive syndromes*	390	7.6	293	9.5	102	10.5	22	9.4
Tubal ligation	220	4.3	73	2.4	107	11.0	36	15.3
Prolonged gestation	172	3.4	143	4.6	20	2.1	8	3.4
Fetal distress and IUGR**	159	3.1	135	4.4	18	1.8	5	2.1
Breech/cormic presentation	131	2.6	100	3.2	25	2.6	0	0.0
Diabetes	83	1.6	63	2.0	11	1.1	6	2.6
Premature placental abruption (PPD)	49	1.0	35	1.1	11	1.1	1	0.4
Placenta previa	31	0.6	22	0.7	6	0.6	0	0.0
Other ***	1428	27.8	1120	36.3	248	25.4	47	20.1
No information	626	12.2	520	16.8	93	9.5	13	5.5

IUGR: intrauterine growth restriction.

Source: *Inquérito Nascer no Brasil* (Birth in Brazil survey).

As observed in Table 4, the probability of VBAC decreased by 75% in the case of a second cesarean section (OR = 0.25, 95%CI 0.14–0.44), and by 82% in the instance of a third cesarean section (OR = 0.18, 95%CI 0.07–0.49). Assistance from the private sector diminished the likelihood of VBAC by 56% (OR = 0.44, 95%CI 0.22–0.88), and experiencing obstetric complications led to a decrease of 38% in the chances of VBAC (OR = 0.62, 95%CI 0.43–0.89). The odds of experiencing VBAC were roughly 4.5 times higher (OR = 4.53, 95%CI 3.01–6.79) for women who were designated for a vaginal birth towards the end of pregnancy. Additionally, the odds were almost 3 times higher (OR = 2.80, 95%CI 1.47–3.13) for those with a history of a prior vaginal birth. Furthermore, women who had labor induced exhibited 5 times higher odds (OR = 5.36, 95%CI 2.74–10.50), whereas those admitted to the hospital with 4 to 5 centimeters of cervical dilation increased by 8 times the odds (OR = 8.10, 95%CI 5.01–13.12). For those admitted with more than 6 centimeters of dilation, the odds were elevated by 13.10 times (OR = 13.10, 95%CI 6.48–30.20). Interestingly, women without a partner displayed a 70% higher chance of experiencing a vaginal birth (OR = 1.70, 95%CI 1.04–2.79). Variables such as age group, educational background, adequacy of prenatal care, and reasons for the previous cesarean section did not exhibit statistically significant differences.

**Table 4:** Factors associated with vaginal births following cesarean sections in Brazil. Period: 2011-2012. Source: Birth in Brazil survey.

Number of cesarean sections	OR	95%CI
Number of cesarean sections — Raw values		
1	1	-
2	0.11	0.18 –0.66
3 or more	0.13	0.04 –0.37
Number of cesarean sections — Adjusted values		
1	1	-
2	0.25	0.14 –0.44
3 or more	0.18	0.07 –0.49
Financing		
Public	1	-
Private	0.44	0.22–0.88
Age (years)		
12–19	1	-
20–34	0.68	0.39–1.89
35 or older	0.54	0.28–1.06
Education (years)		
Up to 7	1	-
8–10	0.96	0.61–1.57
11 or older	0.83	0.55–1.26
Marital Status		
Married/in a relationship	1	-
Single	1.70	1.04–2.79
Prenatal		
No prenatal	1	-
Inadequate	1.13	0.47–3.79
Adequate	1.21	0.44–3.32
Previous vaginal birth		
Yes	2.80	1.97–3.98
Complications		
Yes*	0.62	0.43–0.89
Induction		
Yes	5.36	2.74–10.50
Dilation on admission		
Up to 3 cm	1	-
4–5 cm	8.10	5.01–13.12
Over 6 cm	13.10	6.48-30.20
Decision on the type of delivery at the end of pregnancy		
No preference	1	-
Vaginal birth	4.53	3.01–6.79
C-section	0.25	0.16–0.38
Reason for the previous cesarean section		
Previous cesarean section	0.30	0.09–1.03
Transverse/pelvic baby	1.02	0.43–2.44
Large baby/lack of dilation	0.79	0.57–1.08

OR: odds ratio; 95%CI: 95% confidence interval.

Source: *Inquérito Nascer no Brasil* (Birth in Brazil survey).

* Category encompasses complications or any clinical and obstetric condition potentially related to obstetric emergencies before delivery. This could include issues such as premature placental detachment, diabetes, HIV infection, hypertensive syndrome, or placenta previa.

Note: in bold statistically significant values.

## DISCUSSION

Out of the total of women included in the sample of the Birth in Brazil survey, 20.9% had undergone previous cesarean sections before entering the research, and 85.1% underwent the surgery again, with 75.5% occurring before going into labor. In contrast, the overall study sample showed a cesarean rate of 52%, with 34.1% occurring before labor began^20^. According to the World Health Organization (WHO), the group of women who underwent previous cesarean section accounts for 21.9% of births, and an acceptable rate for repeat surgery would fall between 50% and 60%^21^.

A second cesarean section reduces the likelihood of VBAC by 75%, and having three or more cesarean sections reduces that number by 82%. Data on the situation of the country indicate a strong trend towards repeat cesarean sections, contrary to studies indicating the safety of VBAC and the potential complications, which increase with the number of surgeries^12^. Hence, avoiding the first cesarean section is one of the recommended strategies to reduce cesarean rates and their effects on maternal and child health^7^. The VBAC rate among women who went into labor was 60.8%, a value similar to those found in other studies ranging from 57.6% to 74%^12^.

The magnitude of previous cesarean sections revealed that a higher number of surgeries is related to higher parity. It is important to highlight that 22.3% of women who had undergone three or more cesarean sections also had a prior vaginal birth. This observation could potentially indicate insufficient access to family planning services, a variable that contributes to restricted access to healthcare services and other fundamental rights, often more prevalent among socioeconomically vulnerable groups.

A Brazilian study that compared four cohorts from Pelotas, spanning from 1982 to 2015, revealed that despite a decline in parity across all social strata, black or mixed-race and economically disadvantaged women continue to experience higher parity and shorter intervals between pregnancies. The interaction between income and race in terms of interpregnancy intervals and at least one previous pregnancy demonstrated an increase in inequality over the studied period^22^. Data from the Birth in Brazil study indicated that black women are more susceptible to social inequality, marked by lower educational levels, concentration in lower social classes, and higher rates of inadequate prenatal care^23^.

According to the Ministry of Health, the group who has undergone three or more cesarean sections is relatively smaller, as tubal ligation is frequently conducted after the third surgery. While a vaginal birth in such cases has a limited impact on overall cesarean rates, the heightened risk of uterine rupture in VBA3C (Vaginal Birth After Three or More Cesareans) must be weighed against the potential rise in complications such as hemorrhage, hysterectomy, and injury to the bladder or intestinal loops that could occur when opting for repeat cesarean after multiple surgeries^24^.

The likelihood of VBAC was virtually non-existent within the private sector. In births involving previous cesarean sections that were funded by the public Unified Health System (SUS), one-quarter resulted in vaginal births, accounting for 96.1% of all VBAC cases. In contrast, within the private healthcare system, almost all women with previous cesarean sections underwent repeat cesarean surgeries. An analysis by Nakamura-Pereira et al. demonstrated that even among women who underwent previous cesarean sections who were eligible for a trial of labor after cesarean (TOLAC), 79.4% underwent surgery, with 66.1% being elective. In the private healthcare system, this percentage escalated to 95.3%. This suggests that clinical conditions alone do not necessarily govern the decision to opt for a repeat cesarean section^10^.

The disparities in cesarean rates between public and private health care services are intricate, encompassing the financial and cultural dimensions. In the private sector, interventionist health care model that prioritizes diagnostic procedures and the utilization of advanced surgical skills often overshadows a woman-centered care, which is crucial for vaginal birth monitoring. Factors such as perceived control over the process, scheduling convenience, protection against potential genital harm, and the elevated socioeconomic status associated with these elements can contribute to the higher rates of cesarean sections observed within the private health care system^25^.

In the public sector, while certain private unit practices may be mirrored, the Brazilian Ministry of Health has initiated actions to promote lower cesarean rates, with measures focused on decreasing primary cesarean surgeries^26^.

The Ministry of Health recommends Vaginal Birth After Cesarean (VBAC) for women who underwent 1 or 2 prior cesarean sections and do not have contraindications for vaginal birth^24^. However, even within the public sector, 52.2% of births were elective cesareans, i.e., conducted before the onset of labor. When including cesarean sections performed with ongoing labor, the rate escalates to 70.6%.

The data highlight that the likelihood of VBAC was elevated for women who upheld their decision for vaginal birth until the culmination of pregnancy, had a history of previous vaginal birth, underwent labor induction, or were admitted to the hospital with cervical dilation ranging from 4 to 5 centimeters, with even greater odds for dilation exceeding 6 centimeters.

The findings are consistent with research conducted by D’Orsi *et al*.^8,9^, which assessed factors associated with the probability of VBAC in Brazil based on medical record data from a public maternity hospital in Rio de Janeiro. VBAC was also strongly linked with women who underwent only one prior cesarean, cervical dilation upon admission exceeding 3 cm, a record of at least one prior vaginal birth, and lower levels of education—paralleling the outcomes identified in the present analysis. The authors of the current study also discovered that there was a smaller likelihood of VBAC for women with high blood pressure and gestational age under 37 weeks, even though these factors were not typically included in the standard risk assessment.

Greater dilation and more favorable cervical conditions were also relevant factors in studies by Maroyi *et al*.^29^ in the Democratic Republic of Congo; Fitzpatrick *et al*.^30^, in Scotland; and Mi *et al*.^31^, in China. These studies included women from various nationalities, featuring diverse physical and social backgrounds, along with distinct obstetric cultures. The majority of these studies pointed to the impact of socioeconomic status, non-dominant ethnicity, previous vaginal birth, having undergone only one prior cesarean, and favorable cervical dilation conditions that should ideally align with labor onset before hospital admission.

Women who arrived at the end of their pregnancy with a pre-established inclination towards cesarean delivery exhibited lower odds of VBAC. We must recognize that the choice regarding the type of delivery at the end of pregnancy is heavily influenced by the prenatal care received. According to findings from *Nascer no Brasil*, only 27.6% of Brazilian women reported commencing prenatal care with a preference for surgery. Within the public sector, this inclination was even lower—at 15%—and remained consistent throughout. In contrast, within the private sector, 36.1% initially leaned towards surgery, and by the conclusion of pregnancy, 67.6% had already chosen cesarean delivery. The most notable proportion of women harboring a preference for cesarean at the outset of pregnancy can be found among multiparous within the private system who had previously undergone the procedure, accounting for a significant 73.2% of this subset^32^.

Reasons for the previous cesarean were provided by the women themselves, with the options “Large baby/no passage or dilation/baby did not descend or engage” emerging as the most prevalent rationale during interviews. Although the reason for the prior cesarean did not appear as a significant factor for VBAC in this study, researchers have found that indications of labor dystocia for the previous cesarean might be correlated with diminished chances of VBAC during labor^33^.

Notably, the data for this study were collected between the years 2011 and 2012, nearly a decade prior to the analysis. However, cesarean rates in Brazil remained relatively stable during that period, and substantial shifts in the care provided to this demographic of women are not believed to have taken place. Leal *et al*.^34^ compared the findings from the *Nascer no Brasil* survey within the public health care system with the evaluation studies of *Rede Cegonha*, a program initiated by the Ministry of Health. The findings revealed that the cesarean rate remained relatively unchanged, with a shift from pre-labor cesareans to intrapartum cesareans. When comparing the private health care system with the *Parto Adequado* program, a reduction in cesarean rates was observed, albeit still elevated.

The elevated cesarean rates transition from being a protective measure to a potential risk factor, which signifies an iatrogenic epidemic—where medical intervention inadvertently causes harm^35^. As such, these high cesarean rates become a pressing public health concern. It is crucial to recognize that, beyond the prevention of unnecessary cesarean sections in nulliparous women, the group of women with previous cesareans significantly contributes to the overall rates of cesarean occurrences in Brazil^36,37^_._ To counteract this recurring effect, policies promoting Vaginal Birth After Cesarean (VBAC) must be implemented to prevent women from undergoing multiple cesareans, as they are more susceptible to complications.

## CONCLUSION

The study revealed that the profile of women who underwent previous cesareans in Brazil changed according to the number of cesarean surgeries performed. The escalation in the number of cesareans might be linked to greater parity and increased social inequality. Despite scientific studies indicating the safety of vaginal births after cesareans, the majority of women are directed to another surgery, a significant portion of which happens before the beginning of labor, in public and private health care sectors. This topic requires greater visibility and attention from policymakers to reduce unnecessary cesarean rates in nulliparous women and encourage VBAC to decrease overall cesarean rates and their consequences for maternal and child health.
