# Supplementary material for: Characteristics of women who underwent one or more previous cesarean sections according to Nascer no Brasil
Source: Rev Saude Publica. 2023 Nov 8;57:89. doi: 10.11606/s1518-8787.2023057004819 (PMC10681529; doi:10.11606/s1518-8787.2023057004819)
Supplement: Translation

# Translation: Características de mulheres com uma ou mais cesáreas anteriores no nascer no Brasil

**Authors:** Marina Barreto Alvarenga, Silvana Granado Nogueira da Gama, Marcos Nakamura-Pereira

**Affiliations:** I Brasil Fundação Oswaldo Cruz. Escola Nacional de Saúde Pública Sérgio Arouca. Programa de Epidemiologia em Saúde Pública, Rio de Janeiro, RJ, Brasil; II Brasil Fundação Oswaldo Cruz. Escola Nacional de Saúde Pública Sérgio Arouca. Departamento de Epidemiologia e Métodos Quantitativos em Saúde, Rio de Janeiro, RJ, Brasil; III Brasil Fundação Oswaldo Cruz. Escola Nacional de Saúde Pública Sérgio Arouca. Programa de Epidemiologia em Saúde Pública, Rio de Janeiro, RJ, Brasil; IV Brasil Instituto Fernandes Figueira, Rio de Janeiro, RJ, Brasil

**Keywords:** Parto Obstétrico, Nascimento Vaginal após Cesárea, Parto Normal, Cesárea, Saúde Materna

## Abstract

**OBJETIVO:**

Analisar descritivamente as parturientes brasileiras com cesariana anterior e apontar os fatores associados ao parto vaginal após cesárea (Vaginal Birht After Cesarean– VBAC) no Brasil.

**MÉTODOS:**

Foram utilizados dados de mulheres com uma, duas ou três e mais cesáreas da pesquisa Nascer no Brasil. As diferenças entre categorias foram avaliadas pelo teste de qui-quadrado (χ^2^). As variáveis que apresentaram diferença significativa (< 0,05) foram incluídas em regressão logística.

**RESULTADOS:**

Do total de 23.894 mulheres, 20,9% tinham cesárea anterior. A maior parte (85,1%) foi submetida a outra cesárea, 75,5% antes do início do trabalho de parto. A porcentagem de VBAC foi de 14,9%, uma taxa de sucesso de 60,8%. Mulheres com três cesáreas ou mais apresentaram maior vulnerabilidade social. As chances de VBAC foram maiores entre aquelas decididas pelo parto vaginal no fim da gestação, com parto vaginal anterior, indução de parto, admitidas com mais de 4 centímetros de dilatação e sem companheiro. Assistência no sistema privado, ter duas cesáreas ou mais, complicações obstétricas e decisão por cesariana no final da gestação diminuíram as chances de VBAC. Faixa etária, escolaridade, adequação do pré-natal e razão da cesárea anterior não apresentaram diferença significativa.

**CONCLUSÃO:**

A maior parte das mulheres com cesárea anterior no Brasil é encaminhada para uma nova cirurgia, e o maior número de cesáreas está associado à maior iniquidade social. Os fatores associados ao VBAC foram decisão pelo parto vaginal no fim da gestação, parto vaginal anterior, maior dilatação cervical na internação, indução, atendimento no sistema público de saúde, ausência de complicações obstétricas e ausência de companheiro. São necessárias ações de estímulo ao VBAC, visando reduzir taxas globais de cesarianas e suas consequências para a saúde materno-infantil.

## INTRODUÇÃO

No Brasil, desde 2009^1^a maioria dos nascimentos ocorre por cesariana. A despeito de todas as políticas públicas implementadas no país nos últimos anos^2^, dados do Ministério da Saúde apontam que, em 2020, a taxa nacional foi de 57,2%^6^. Segundo a Organização Mundial da Saúde (OMS), taxas de cesárea maiores que 10% a 15% não estão associadas com redução de mortalidade materna e neonatal, não havendo evidências de benefícios da cirurgia em mulheres sem indicação clínica. Além disso, o procedimento cirúrgico acarreta mais riscos imediatos e a longo prazo para a saúde das mulheres e de seus filhos, inclusive em possíveis gestações futuras^7^.

Se para as mulheres que estão grávidas pela primeira vez as chances de um parto vaginal no Brasil já não são grandes, para as mulheres com cesarianas anteriores essas chances são remotas^7^. A ideia de “uma vez cesárea, sempre cesárea”, apesar de ser datada de 1916^10^, permanece na cultura obstétrica do país a despeito de evidências científicas que demonstram segurança no parto vaginal após cesariana (Vaginal Birth After Cesarean – VBAC). A decisão sobre o tipo de parto nas gestações subsequentes a uma cesariana torna-se uma questão importante, pois existem riscos associados a repetidas cirurgias no mesmo local, como a maior probabilidade de aderências, hemorragias e placentação anormal (placenta prévia e acreta)^11^, que podem culminar em desfechos maternos graves, incluindo óbito.

No Brasil, há poucos dados disponíveis a respeito da saúde materna e perinatal após cesarianas, sendo necessárias mais pesquisas para a compreensão do tema. O objetivo do estudo é fazer uma análise descritiva das parturientes brasileiras com cesariana anterior em uma gestação subsequente, bem como apontar os fatores associados ao VBAC no Brasil.

## MÉTODOS

O presente estudo utiliza dados do Nascer no Brasil, inquérito nacional sobre parto e nascimento, composto por mulheres no pós-parto e seus recém-nascidos, realizado no período de fevereiro de 2011 a outubro de 2012, com amostra representativa das cinco macrorregiões do país, residentes de capitais e não capitais, em serviços privados, públicos e mistos. Os dados foram coletados por meio de entrevistas durante a internação hospitalar, extração de dados dos prontuários, cartões de pré-natal (quando disponíveis) e duas ligações telefônicas após a alta hospitalar, a partir de uma amostragem complexa com 266 hospitais^14,15^.

Este estudo tem um recorte de 4.987 mulheres com cesárea anterior, sendo excluídas, portanto, todas as nulíparas e as multíparas com antecedente de apenas partos vaginais (Figura). Foram avaliadas as características sociodemográficas e obstétricas, com estratificação por quantidade de cirurgias em uma, duas ou três e mais cesáreas e por tipo de parto na gestação atual, sendo ele vaginal (VBAC) ou cesárea de repetição.

**Figura:** Fluxograma de mulheres com uma ou mais cesáreas do estudo Nascer no Brasil. Período: 2011–2012. Fonte: Inquérito Nascer no Brasil.

VBAC: Vaginal Birth After Cesarean.

Foram selecionadas como variáveis sociodemográficas: região de ocorrência (Norte, Nordeste, Centro-Oeste, Sudeste e Sul); localização da cidade (capital, interior); financiamento para o parto (SUS, privado); faixa etária (12–19 anos, 20–34 anos, > 35 anos); raça/ cor segundo o Instituo Brasileiro de Geografia e Estatística (IBGE) (branca, preta, parda, amarela, indígena); anos de escolaridade (≤ 7, 8–10, ≥ 11), situação conjugal (com e sem companheiro) e classificação econômica agrupada (A/B, C ou D/E) segundo a Associação Brasileira de Empresas de Pesquisa (ABIPEME^16^.

Os fatores obstétricos selecionados foram: tipo de parto na gestação atual (vaginal, cesárea); cesárea de acordo com o trabalho de parto (cesárea sem trabalho de parto, cesárea intraparto); parto vaginal anterior; mesmo profissional no pré-natal e no parto (sim, não); idade gestacional (prematuro: ≤ 36 semanas, termo: 37–41 semanas, pós-termo: ≥ 42 semana); indução de trabalho de parto (induzido, espontâneo); preferência de parto no início da gestação e preferência de parto no final da gestação (vaginal, cesárea, não tinha preferência).

A adequação do pré-natal (sem pré-natal, inadequado e adequado) foi baseada no estudo de Domingues et al.^17^. O pré-natal foi considerado adequado quando iniciado até 12 semanas de gestação, com o mínimo de seis consultas, registro de pelo menos um resultado de cada exame da rotina pré-natal em cartão da gestante e orientação à maternidade de referência fornecida.

Com relação à dilatação cervical no momento da internação (£ 3 centímetros, 4–5 centímetros, ≥ 6 centímetros), verificou-se que 95% das mulheres sem essa informação em prontuário tiveram cesárea antes do trabalho de parto. Essas foram consideradas, então, na categoria dilatação menor do que 3 centímetros. Os 5% restantes estavam distribuídos de maneira igualitária entre as outras categorias. Para a taxa de sucesso do trabalho de parto foi considerada a porcentagem de partos normais entre as mulheres que entraram em trabalho de parto.

Foram consideradas emergências ou complicações obstétricas: síndromes hipertensivas, diabetes ou alguma outra condição clínica e obstétrica potencialmente relacionada a emergências obstétricas antes do parto, que podem ter sido descolamento prematuro de placenta, placenta prévia, infecção pelo HIV, ou outro tipo^9,18^. Os dados foram coletados em prontuário, com exceção de raça/cor, escolaridade, situação conjugal, perfil econômico e razão da última cesárea. A adequação pré-natal considerou os dados de cartão pré-natal. A indicação da cesárea atual incluiu até três opções para cada paciente, segundo o obstetra.

Foi realizada uma análise estatística descritiva para a apresentação de frequências absolutas e relativas das variáveis sociodemográficas e obstétricas, de acordo com o número de cesáreas anteriores e o tipo de parto na gestação atual. As diferenças entre as categorias foram avaliadas pelo teste de qui-quadrado (χ^2^), considerando um valor de p < 0,05 para significância estatística. As variáveis que apresentaram diferença estatisticamente significativa nas análises descritivas foram incluídas no modelo linear generalizado de maneira hierarquizada, iniciando pelas características sociodemográficas e, em seguida, incluindo as características obstétricas. O poder do efeito de tais variáveis poderia se anular no ajuste estatístico. Foi utilizado o software R, versão 1.2.1335, para as análises.

### Aspectos Éticos

O estudo foi aprovado pelo Comitê de Ética da Escola Nacional de Saúde Pública Sergio Arouca, Fundação Oswaldo Cruz (ENSP/Fiocruz), sob o CAAE 50697621.4.0000.5240, com número de parecer: 4.950.262.

## RESULTADOS

Do total de 23.894 mulheres incluídas no Nascer no Brasil, 4.987 (20,9%) tinham ao menos uma cesariana prévia (Figura). Na Tabela 1, evidencia-se que a maior parte das mulheres tiveram o atendimento financiado pelo Sistema Único de Saúde (SUS) (71,8%), tinham de 20 a 34 anos (76,8%), se classificavam como pardas (52,4%), tinham mais de 11 anos de escolaridade (51,6%), viviam com companheiro (86,6%) e pertenciam à classe econômica C (49,3%).

**Tabela 1:** Tabela de frequência. Características sociodemográficas das mulheres com cesárea anterior no Brasil. Distribuição por quantidade de cesáreas anteriores, tipo de parto na gestação atual e porcentagens de VBAC por categoria. Período: 2011-2012. Fonte: Inquérito Nascer no Brasil.

Fatores sociodemográficos	Todas as mulheres com cesárea anterior		Com 1 cesárea anterior		Com 2 cesáreas anteriores		Com ≥3 cesáreas anteriores		Valor de p	VBAC		% VBAC	Cesáreas de repetição		Valor de p
					
	n	%	n	%	n	%	n	%		n	%		n	%	
Total	4.987	100	3.817	100	948	100	222	100		743	100	14,9	4.246	100	
Região															
Norte	426	8,6	313	8,2	104	10,9	10	4,5		83	11,2	19,5	343	8,1	
Nordeste	1.194	24	952	25	219	23,1	22	9,8		148	19,9	12,4	1.045	24,6	
Centro-Oeste	368	7,4	256	6,7	84	8,9	27	12,4	< 0,001	47	6,4	12,8	320	7,6	0,15
Sudeste	2.281	45,7	1.747	45,8	418	44,1	117	52,5		343	45,3	15	1.938	45,6	
Sul	718	14,4	549	14,4	123	13	46	20,7		120	16,2	16,7	598	14,1	
Cidade															
Capital	1.831	36,7	1.391	36,4	354	37,4	86	38,7	0,78	309	41,6	16,9	1.523	64,1	0,13
Interior	3.156	63,3	2.426	63,6	592	62,6	136	61,3		434	58,4	13,8	2.723	35,9	
Hospital															
SUS*	3.584	71,8	2.629	68,9	758	79,9	196	88,2	< 0,001	713	96,1	24,6	2.871	67,6	
Privado	1.404	28,2	1.118	31,1	190	21,1	26	11,8		29	3,9	2	1.375	32,4	
Faixa etária (anos)															
12–19	252	5,1	224	5,9	22	2,3	6	2,6		60	8,1	23,8	192	4,5	
20–34	3.834	76,8	2.933	76,8	740	78	161	71,7	< 0,001	602	81	15,7	3.233	76,1	< 0,001
≥ 35	901	18,1	660	17,3	186	29,7	55	25,7		81	10,9	9	821	19,4	
Raça/cor															
Branca	1.935	38,8	1.520	39,8	332	35	82	37,1		253	34,1	13,1	1.682	39,6	
Preta	375	7,5	286	7,5	62	6,6	27	12,1		70	9,4	18,7	305	7,2	
Parda/Morena	2.615	52,4	1.966	51,5	536	56,5	112	50,6	0,02	407	54,8	15,6	2.208	52	0,12
Amarela	53	1,1	36	0,9	16	1,7	1	0,3		10	1,3	18,9	43	1	
Indígena	11	0,2	9	0,2	2	0,3	0	0		3	0,5	27,3	8	0,2	
Escolaridade (anos)															
≤ 7	1.281	25,7	847	22,2	321	33,9	113	50,8		290	38,9	22,6	993	23,4	
8–10	1.134	22,7	827	21,7	251	26,5	56	25,4	< 0,001	207	27,9	18,3	927	21,8	< 0,001
≥ 11	2.572	51,6	2.143	56,1	376	39,6	53	23,8		246	33,2	9,6	2.326	54,8	
Situação conjugal															
Sem companheiro	669	13,4	477	12,5	151	16	40	18	0,04	150	20,3	22,4	518	12,2	< 0,001
Com companheiro	4.317	86,6	3.337	87,5	797	84	182	82		590	79,7	13,7	3.272	87,8	
Classe econômica segundo ABIPEME															
Classe A+B	1.621	32,5	1.306	33,2	259	27,3	56	25,1		117	15,7	7,2	1.505	35,5	
Classe C	2.457	49,3	1.861	48,8	481	50,8	115	51,6	< 0,001	424	57	17,3	2.035	47,9	<0,001
Classe D+E	909	18,2	649	17	208	21,9	51	23,3		202	27,3	22,2	706	16,6	

SUS: Sistema Único de Saúde; ABIPEME: Associação Brasileira de Empresas de Pesquisa; VBAC: Vaginal Birth After Cesarean.

Fonte: Inquérito Nascer no Brasil.

Algumas categorias apresentaram aumento em suas proporções conforme crescia o número de cirurgias. As mulheres pretas representavam 7,5% daquelas com 1 cesárea anterior, atingindo 12% entre aquelas com 3 ou mais. A faixa de escolaridade mais baixa, até 7 anos de estudo, eleva-se de 22,2% para 50,8%, e o financiamento pelo SUS aumenta de 68,9 para 88,2%. Os percentuais de mulheres com mais de 35 anos, sem companheiro e pertencentes às classes C, D e E também aumentam.

Considerando todas as mulheres, o percentual de VBAC foi de 14,9%. O SUS foi responsável por 96,1% dos casos de VBAC, e, entre seus partos com histórico de cesárea anterior, 24,6% foram vaginais. Região, tipo de cidade (capital ou não) e raça/cor não apresentaram diferença estatisticamente significativa com relação ao tipo de parto. A avaliação por categoria revelou maiores proporções de VBAC entre as mulheres da região Norte (19,5%), moradoras de capitais (16,9%), indígenas (27,3%), com faixa etária de 12 a 19 anos (23,8%), com menos de 7 anos de escolaridade (22,6%), nas classes econômicas C e D (22,2%) e sem companheiro (22,4%).

Na Tabela 2 são apresentadas as características obstétricas das mulheres com cesárea anterior. A maior parte delas tinha uma cesárea (76,5%), destas, 81,3% foram submetidas a outra cesárea, sendo 75,5% antes do trabalho de parto. O percentual de cesárea na atual gestação foi de 97,0% quando havia 2 ou mais cirurgias anteriores.

**Tabela 2:** Tabela de frequência. Características obstétricas das mulheres com cesárea anterior no Brasil. Distribuição por quantidade de cesáreas anteriores, tipo de parto na gestação atual e porcentagens de VBAC por categoria. Período: 2011-2012. Fonte: Inquérito Nascer no Brasil.

Fatores sociodemográficos	Todas as mulheres com cesárea anterior		Com 1 cesárea anterior		Com 2 cesáreas anteriores		Com ≥ 3 cesáreas anteriores		Valor de p	VBAC		% VBAC	Cesáreas de repetição		Valor de p
					
	n	%	n	%	n	%	n	%		n	%		n	%	
Total	4.987	100	3817	100	948	100	222	100		743	100	14,9	4.246	100	
Número de cesáreas															
1	3.817	76,5	-	-	-	-	-	-	-	714	96	18,7	3.104	73,1	
2	948	19	-	-	-	-	-	-	-	23	3,1	2,4	926	21,8	< 0,001
≥ 3	222	4,5	-	-	-	-	-	-	-	6	0,9	2,7	216	5,1	
Cesárea de acordo com trabalho de parto															
Cesárea sem trabalho de parto	3.768	75,5	2.723	71,3	844	89	200	90.0	< 0,001	-	-	-	3.768	88,7	
Cesárea intraparto	478	9,6	380	10	81	8,9	16	7,2		-	-	-	478	11,3	-
Parto vaginal anterior															
Sim	1049	21	840	22	159	16,8	22	22,3	0,04	412	55,6	55,5	636	15,1	< 0,001
Não	3932	79	2971	73	789	83,2	78	77,7		328	44,4		3.604	84,9	
Taxa de sucesso de trabalho de parto*															
Partos vaginais	743	60,8	714	65,2	23	22,1	6	28,1	-						
Pré-natal															
Sem pré-natal	63	1,3	39	1	12	1,3	10	4,9		18	2,4	27,2	45	1,1	
Inadequado	2.146	43	1.595	41,8	441	46,5	110	49,7	< 0,001	404	54,4	18,2	1.743	41	< 0,001
Adequado	2.779	55,7	2.183	57,2	495	52,2	101	45,4		321	43,2	11,2	2.458	57,9	
Mesmo profissional no pré-natal e no parto															
Sim	1.716	34,4	1.378	36,1	293	30,9	44	20,1	< 0,001	31	4,2	1,8	1.685	39,7	
Não	3.271	65,6	2.439	63,9	655	69,1	178	79,9		711	95,8	21,7	2.560	60,3	< 0,001
Idade gestacional (semanas)															
Prematuro ( ≤ 36)	491	9,9	354	9,3	110	11,6	27	12,2		77	10,4	15,7	413	9,7	
Termo (37–41)	4.397	88,1	3.377	88,4	827	87,3	190	85,4	0,15	647	87,2	14,7	3.750	88,3	0,84
≥ 42	102	2	86	2,3	11	1,1	5	2,4		18	2,7	17,6	84	2	
Presença de complicações obstétricas															
Hipertensão arterial	665	13,3	499	13,1	127	13,4	39	17,8	0,24	53	7,2	8	612	14,4	< 0,001
Diabetes	538	10,8	404	10,6	100	10,5	34	15,3	0,29	65	8,8	12,1	473	11,1	0,17
Emergências ou complicações**	1.183	23,7	889	23,3	222	23,4	72	32,3	0,05	113	15,3	9,6	1.070	25,2	< 0,001
Início de trabalho de parto***															
Induzido	212	4,2	208	5,4	1	0,1	3	1,4	< 0,001	106	14,3	50	106	2,4	< 0,001
Espontâneo	1.109	22,2	986	25,8	104	11,1	19	8,6		636	85,6	57,3	473	11,2	
Dilatação na internação (cm)															
≤ 3	4.271	85,6	3.180	83,3	880	92,8	211	95		316	42,8	7	3.955	93,1	
4–5	416	8,4	367	9,6	40	4,2	9	4,1	< 0,001	223	29,9	54	193	4,6	< 0,001
≥ 6	300	6	270	7,1	28	3	2	0,9		203	27,3	68	97	2,3	
Preferência pelo tipo de parto															
Parto vaginal	1.756	35,2	1.426	37,4	254	26,9	74	33,7		404	54,4	23	1.352	31,8	
Cesárea	2.869	57,5	2.114	55,4	636	67	118	54,4	< 0,001	292	39,4	10,2	2.576	60,7	< 0,001
Não tinha preferência	364	7,3	277	7,2	58	6,1	29	12,9		46	6,2	12,6	317	7,5	
Decisão no final da gestação															
Parto vaginal	605	12,1	572	15	30	3,2	3	1,3		373	50,5	61,6	232	12,1	
Cesárea	3.239	64,9	2.247	58,9	798	84,1	194	87,3	< 0,001	90	12,1	2,8	3.150	64.9	< 0,001
Não tinha preferência	1.143	22,9	998	26,2	120	12,7	25	11,4		280	37,7	25,1	864	22.9	

VBAC: Vaginal Birth After Cesarean.

Fonte: Inquérito Nascer no Brasil.

*Traz a porcentagem baseada no total de mulheres que entraram em trabalho de parto.

** Complicações ou alguma condição clínica e obstétrica potencialmente relacionada a emergências obstétricas antes do parto, que podem ter sido descolamento prematuro de placenta, diabetes, infecção pelo o HIV ou de qualquer outro tipo, síndrome hipertensiva ou placenta prévia.

*** Excluídas dessa análise as mulheres que não entraram em trabalho de parto.

O percentual de partos vaginais entre aquelas que entraram em trabalho de parto foi de 60,8%, o que foi considerado a taxa de sucesso de VBAC na amostra (Figura). Mulheres internadas em trabalho de parto ativo tiveram mais VBAC, seja com 4 a 5 centímetros de dilatação cervical (54%) ou mais de 6 centímetros de dilatação cervical (68%). 55,5% das mulheres com VBAC, além da cesariana, tinham parto vaginal anterior.

A maior parte das mulheres referiu desejar outra cesárea no início da gestação (57,5%), valor ainda maior entre as mulheres com 2 cesáreas (67%). Em todos os grupos houve aumento na decisão pela cesárea ao final da gestação, chegando a 64,9% no total. A categoria com maior porcentagem de VBAC no estudo foi a de mulheres que mantiveram a preferência pelo parto vaginal até o final do pré-natal (61,6%).

O grupo de mulheres com 3 ou mais cesáreas apresentou maior frequência de gestações sem pré-natal (4,9%), ou pré-natal inadequado (49,7%) e com emergências ou complicações (32,3%). Esse grupo também teve menor porcentagem do mesmo profissional no pré-natal e no parto (20,1%), mais cesáreas de repetição sem trabalho de parto (90%) e, consequentemente, as internações com menos de 3 cm de dilatação (95%).

As mulheres foram questionadas quanto a razão da última cesárea realizada, e a “falta de passagem ou dilatação, e bebê grande ou que não encaixou/desceu” foi o motivo mais prevalente (42,7%), seguido por pressão alta (10,5%), apresentação pélvica/córmica (8%), medo da dor do parto (4,7%), e gestação prolongada (4,3%). As indicações para a cesárea atual, segundo dados do médico obstetra no prontuário, estão descritas na Tabela 3. A cesárea anterior foi citada como motivo para a cirurgia atual em 44,1% dos casos, chegando a 73,6% entre as mulheres com três cesáreas anteriores. As demais razões mais prevalentes foram a desproporção céfalo-pélvica (9,5%) e síndromes hipertensivas (7,6%).

**Tabela 3:** Razões apontadas pelo obstetra para cesárea na gestação atual em mulheres com cesárea anterior no Brasil. Período: 2011-2012.

Razões para a cesárea	Todas as mulheres com cesárea anterior		Com 1 cesárea anterior		Com 2 cesáreas anteriores		Com ≥ 3 cesáreas anteriores	
			
	n	%	n	%	n	%	n	%
Cesariana anterior	2.256	44,1	1.398	45,2	683	70,1	173	73,6
Desproporção céfalo pélvica	490	9,5	452	14,6	25	2,6	6	2,6
Síndromes hipertensivas*	390	7,6	293	9,5	102	10,5	22	9,4
Laqueadura	220	4,3	73	2,4	107	11	36	15,3
Gestação prolongada	172	3,4	143	4,6	20	2,1	8	3,4
Sofrimento fetal e RCIU**	159	3,1	135	4,4	18	1,8	5	2,1
Apresentação pélvica/ córmica	131	2,6	100	3,2	25	2,6	0	0
Diabetes	83	1,6	63	2	11	1,1	6	2,6
Descolamento prematuro de placenta (DPP)	49	1	35	1,1	11	1,1	1	0,4
Placenta prévia	31	0,6	22	0,7	6	0,6	0	0
Outros ***	1.428	27,8	1.120	36,3	248	25,4	47	20,1
Sem informação	626	12,2	520	16,8	93	9,5	13	5,5

Fonte: Inquérito Nascer no Brasil.

RCIU: restrição de crescimento intrauterino.

*Hipertensão/Pré-eclâmpsia/Eclâmpsia e síndrome HELLP.

**Incluído em outros além das respostas abertas que não puderam ser reclassificadas, pouco líquido amniótico, laqueadura, diabetes e outras situações.

Na Tabela 4, verifica-se que a chance de VBAC diminuiu 75% na segunda cesárea (OR = 0,25, 95%IC 0,14–0,44), e 82% na terceira cesárea (OR = 0,18, 95%IC 0,07–0,49). Ser atendida no setor privado reduziu a chance de VBAC em 56% (OR =0,44, 95%IC 0,22–0,88), e ter complicações obstétricas em 38% (OR = 0,62, 95%IC 0,43–0,89). A chance de um VBAC foi aproximadamente 4,5 vezes (OR = 4,53, 95%IC 3,01–6,79) maior para mulheres que estavam decididas por um parto vaginal no final da gestação; para as que já tinham parto vaginal anterior, quase 3 vezes (OR = 2,80, 95%IC 1,47–3,13); com indução de parto, 5 vezes (OR = 5,36, 95%IC 2,74–10,50); as que foram admitidas no hospital com dilatação de 4 a 5 centímetros, 8 vezes (OR = 8,10, IC 5,01–13,12); e 13,10 vezes para as que foram admitidas com mais de 6 centímetros (OR = 13,10, 95%IC 6,48–30,20). Mulheres sem companheiro tiveram 70% mais partos vaginais (OR = 1,70, 95%IC 1,04– 2,79). A faixa etária, escolaridade, adequação do pré-natal e razão da cesárea anterior não apresentaram diferença estatisticamente significativa.

**Tabela 4:** Fatores associados ao parto vaginal após cesárea no Brasil. Período: 2011-2012.

Número de cesáreas	OR	IC 95%
Quantidade de cesáreas – Valores brutos		
1	1	-
2	0,11	0,18–0,66
3 ou mais	0,13	0,04–0,37
Quantidade de cesáreas -Valores ajustados		
1	1	-
2	0,25	0,14–0,44
3 ou mais	0,18	0,07–0,49
Financiamento		
Público	1	-
Privado	0,44	0,22–0,88
Idade (anos)		
12–19	1	-
20–34	0,68	0,39–1,89
≥ 35	0,54	0,28–1,06
Escolaridade (anos)		
≤ 7	1	-
8–10	0,96	0,61–1,57
≥ 11	0,83	0,55–1,26
Situação conjugal		
Com companheiro	1	-
Sem companheiro	1,7	1,04–2,79
Pré-natal		
Não fez	1	-
Inadequado	1,13	0,47–3,79
Adequado	1,21	0,44–3,32
Parto vaginal anterior		
Sim	2,8	1,97–3,98
Complicações		
Sim*	0,62	0,43–0,89
Indução		
Sim	5,36	2,74–10,50
Dilatação na internação (cm)		
≤ 3	1	-
4–5	8,1	5,01–13,12
≥ 6	13,1	6,48–30,20
Decisão do tipo de parto no final da gestação		
Não tinha preferência	1	-
Parto vaginal	4,53	3,01–6,79
Cesárea	0,25	0,16–0,38
Razão para cesárea anterior		
Cesárea anterior	0,3	0,09-1,03
Bebê transverso/pélvico	1,02	0,43-2,44
Bebê grande /falta de dilatação	0,79	0,57-1,08

OR: odds ratio; IC95%: intervalo de confiança de 95%.

Fonte: Inquérito Nascer no Brasil.

*Complicações ou alguma condição clínica e obstétrica potencialmente relacionada a emergências obstétricas antes do parto, que podem ter sido descolamento prematuro de placenta, diabetes, infecção pelo o HIV ou de qualquer outro tipo, síndrome hipertensiva ou placenta prévia.

Nota: em negrito valores estatisticamente significativos.

## DISCUSSÃO

Do total de mulheres incluídas na amostra do estudo Nascer no Brasil, 20,9% tinham cesarianas prévias à gestação de entrada na pesquisa, e 85,1% passaram novamente pela cirurgia, sendo 75,5% antes de entrar em trabalho de parto, enquanto a amostra total do estudo apresentou 52% de cesáreas, com 34,1% antes do trabalho de parto^19^. Para a Organização Mundial de Saúde (OMS) o grupo de mulheres com cesárea anterior é responsável por 21,9% dos nascimentos, e sua taxa aceitável para uma cirurgia de repetição ficaria entre 50% e 60%^20^.

A segunda cesárea reduz em 75% a chance de um VBAC, e ter três ou mais cesáreas em 82%. Os dados brasileiros apontam uma forte tendência para a cesárea de repetição, em detrimento dos estudos que apontam a segurança dos VBAC e as possíveis complicações, que aumentam com o número de cirurgias^11^. Por isso, evitar a primeira cesárea é um dos caminhos recomendados para a diminuição das taxas de cesariana e seus efeitos na saúde materno-infantil^7^. A taxa de VBAC entre as mulheres que entraram em trabalho de parto foi de 60,8%, valor próximo aos encontrados em outros estudos, que ficaram entre 57,6% e 74%^11^.

A magnitude de cesáreas anteriores revelou que o maior número de cirurgias está relacionado à maior paridade total. Vale ressaltar que 22,3% das mulheres com três cesarianas ou mais também tinham parto vaginal anterior, fato que pode indicar falta de acesso aos serviços de planejamento familiar, uma das variáveis que compõem a falta de acesso aos serviços de saúde, bem como a outros direitos fundamentais, mais frequentes em camadas socioeconômicas mais vulneráveis.

Um estudo brasileiro comparou quatro coortes de Pelotas, com dados de 1982 a 2015, e revelou que, apesar da redução de paridade em todas as camadas sociais, as mulheres pretas ou pardas e pobres continuam com uma maior paridade e menor intervalo entre as gestações. A interação entre renda e cor da pele no intervalo interpartal e pelo menos uma gestação anterior revelou que a iniquidade aumentou durante o período estudado^21^. Dados do Nascer no Brasil já demonstraram que as mulheres pretas estão mais suscetíveis à iniquidade social, com menor escolaridade, concentração nas classes econômicas mais baixas, e apresentam maiores taxas de pré-natal inadequado^22^.

Para o Ministério da Saúde, o grupo com três cesáreas ou mais é menor, uma vez que é comum a realização de ligadura tubária na terceira cirurgia. Apesar de um parto vaginal nesses casos ter pouco impacto nas taxas globais de cesariana, o maior risco de rotura uterina em caso de VBA3C deve ser pesado contra o aumento de hemorragia, histerectomia, lesão vesical de alças intestinais quando se repete um cesariana após múltiplas cirurgias^23^.

A chance de um VBAC foi quase nula no setor privado. Dos partos com cesárea anterior financiados pelo SUS, um quarto foi vaginal, porcentagem que representou 96,1% dos VBAC. No sistema privado de saúde, quase a totalidade das mulheres com cesárea prévia tiveram cesarianas de repetição. Nakamura-Pereira e colaboradores identificaram que, mesmo em mulheres com cesárea anterior elegíveis ao trabalho de parto após cesárea prévia (TOLAC), 79,4% foram submetidas à cirurgia, sendo 66,1% de maneira eletiva. No sistema privado a porcentagem foi de 95,3%, o que aponta que, em geral, não são condições clínicas que estão envolvidas na decisão de realizar uma nova cesariana^9^.

A diferença nas taxas de cesárea entre os serviços públicos e privados são multicausais, com aspectos financeiros e culturais envolvidos. Na rede privada, o modelo intervencionista de atenção à saúde, os diagnósticos e o uso de habilidades cirúrgicas sofisticadas são mais valorizados do que a atenção centrada na mulher, exigida para o acompanhamento de um parto normal. A aparente possibilidade de controle do processo, agendamento, a proteção de um possível dano genital e o maior status socioeconômico atrelado a esses fatores podem influenciar as maiores taxas no sistema privado^24^.

Na rede pública, ainda que se reproduzam atitudes características das unidades privadas, algumas ações do Ministério da Saúde buscaram incentivar a diminuição das taxas de cesariana, além de medidas com intuito de reduzir a cirurgia primária^25^.

O Ministério da Saúde recomenda o VBAC para mulheres com 1 ou 2 cesáreas anteriores, que não apresentem contraindicações ao parto vaginal^23^. Contudo, mesmo no setor público, 52,2% dos partos foram cesarianas eletivas, ou seja, antes do início do trabalho de parto. Contando com aqueles em que a cirurgia foi realizada após seu início, a taxa chega a 70,6%.

Os dados do estudo apontaram que as chances de um VBAC foram maiores para mulheres que persistiram até o final da gestação com a decisão pelo parto vaginal, já tinham um parto vaginal anterior, tiveram indução de parto ou foram admitidas no hospital com 4–5 centímetros de dilatação, sendo ainda maiores com dilatação superior a 6 centímetros.

Os achados vão ao encontro de D’Orsi et al.^7,8^, que avaliaram os fatores associados à chance de um parto vaginal após cesariana no Brasil com base em registros médicos de prontuários de mulheres que deram à luz em uma maternidade pública do município do Rio de Janeiro. O VBAC também esteve mais associado a mulheres com apenas uma cesárea anterior, dilatação cervical na admissão acima de 3 cm, história de pelo menos um parto vaginal anterior e menor escolaridade, dados similares aos encontrados na presente análise. Os autores ainda encontraram menor chance para mulheres com hipertensão arterial e idade gestacional inferior a 37 semanas, que não foram mensurados em nosso recorte de risco habitual.

Maior dilatação e condições de colo uterino mais favoráveis também foram condições relevantes nos estudos de Maroyi et al.^28^, na República Democrática do Congo, Fitzpatrick et al.^29^, na Escócia, e Mi et al.^30^, na China. Os estudos citados contemplam mulheres de diversas nacionalidades, e, com isso, diversas composições físicas e sociais, bem como diferentes culturas obstétricas. A maior parte deles aponta para a influência do nível socioeconômico, da etnia não dominante, do parto vaginal anterior, presença de apenas uma cesárea e das condições favoráveis de dilatação cervical, que devem estar associadas ao início do trabalho de parto, antes da admissão hospitalar.

Mulheres que chegaram ao final da gestação, decididas por uma cesárea, tiveram chances menores de VBAC. Vale ressaltar que a decisão pelo tipo de parto no fim da gestação é influenciável pela assistência pré-natal. Segundo publicação do Nascer no Brasil, apenas 27,6% das mulheres brasileiras referiram iniciar o pré-natal com o desejo pela cirurgia. No setor público, essa preferência é menor, de 15%, e assim permanece até o final, enquanto, no setor privado, 36,1% preferem a cirurgia no início e, ao final da gestação, 67,6% delas já definiram a cesariana como via de parto. A maior proporção de mulheres com preferência pela cesárea no início da gestação está no grupo das multíparas do sistema privado que já passaram pelo procedimento, 73,2%^31^.

A razão da cesárea anterior foi apresentada pelas mulheres, sendo mais prevalente a justificativa apresentada na opção “Bebê grande/não teve passagem ou dilatação/bebê não desceu ou não encaixou” da entrevista. Apesar de a razão da cesárea anterior não aparecer como um fator significativo para o VBAC, pesquisadores já apontaram que indicação de distocia de parto para cesárea anterior podem ser um fator relacionado à diminuição das chances de VBAC trabalho de parto^32^.

Os dados do estudo foram coletados entre os anos de 2011 e 2012, quase 10 anos antes de sua análise, o que pode ser uma limitação do estudo. Entretanto, as taxas de cesariana durante essa década, no Brasil, pouco se alteraram, e não se acredita que tenham ocorrido mudanças substanciais na atenção ao parto desse grupo de mulheres. Leal et al.^33^ compararam os achados da rede pública do Nascer no Brasil com os estudos de avaliação da Rede Cegonha, programa do Ministério da Saúde, e verificaram que a taxa de cesariana não foi alterada, havendo uma substituição das cesáreas antes do trabalho de parto pelas cesáreas intraparto. A comparação da rede privada com o Parto Adequado apontou redução nas taxas de cesárea, que ainda assim permanecem altas.

As altas taxas de cesárea se tornam um problema de saúde pública quando a intervenção, a princípio concebida como um instrumento de proteção, se torna um fator de risco, caracterizando uma epidemia iatrogênica^34^. Por isso, além da prevenção de cesáreas sem indicação clínica em mulheres nulíparas, é importante compreender que o grupo de mulheres com cesáreas anteriores é o que mais contribui para a taxa de cesariana global do Brasil^35,36^_._ E para reverter esse efeito recorrente das cesáreas é necessário que sejam empregadas políticas de estímulo ao VBAC, a fim de evitar mulheres com múltiplas cesáreas, uma vez que elas estão ainda mais propensas a complicações.

## CONCLUSÃO

O estudo revelou que o perfil das mulheres com cesárea anterior no Brasil se altera de acordo com o número de cirurgias realizadas, e o maior número de cesáreas pode estar associado à maior paridade e iniquidade social. Apesar dos estudos apontarem a segurança dos partos vaginais após cesariana, a maior parte das mulheres é encaminhada a uma nova cirurgia, cuja grande maioria ocorre antes do trabalho de parto, tanto no setor público quanto no privado. O tema precisa de maior visibilidade e atenção dos formuladores de políticas públicas, no intuito de reduzir as taxas de cesáreas sem indicação clínica em nulíparas e estimular o VBAC, para a diminuição das taxas globais de cesarianas e suas consequências para a saúde materno-infantil.
